# 4-[(*E*)-(4-Fluoro­benzyl­idene)amino]­phenol

**DOI:** 10.1107/S1600536811019921

**Published:** 2011-06-04

**Authors:** Li-Xia Sun, Yun-Dan Yu, Guo-Ying Wei

**Affiliations:** a College of Materials Science & Engineering, China Jiliang University, Hangzhou 310053, People’s Republic of China

## Abstract

In the title compound, C_13_H_10_FNO, the dihedral angle between the aromatic rings is 55.60 (8)°. In the crystal, mol­ecules are linked by O—H—N hydrogen bonds, forming zigzag *C*(7) chains propagating in [101].

## Related literature

For a related structure and background references, see: Sun *et al.* (2011 ▶). For related structures, see: Nie *et al.* (2008 ▶); Fun *et al.* (2008 ▶); Alhadi *et al.* (2008 ▶).
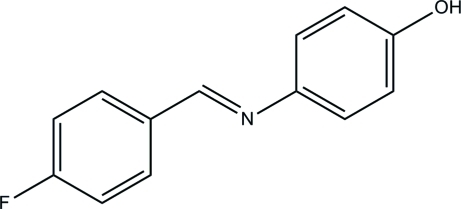


## Experimental

### 

#### Crystal data

C_13_H_10_FNO
*M* *_r_* = 215.22
Monoclinic, 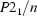

*a* = 9.400 (5) Å
*b* = 12.213 (7) Å
*c* = 9.450 (5) Å
β = 104.666 (5)°
*V* = 1049.5 (10) Å^3^
*Z* = 4
Mo *K*α radiation
μ = 0.10 mm^−1^
*T* = 296 K
0.25 × 0.23 × 0.22 mm

#### Data collection

Bruker APEXII CCD diffractometer
Absorption correction: multi-scan (*SADABS*; Bruker, 2004 ▶) *T*_min_ = 0.976, *T*_max_ = 0.979
5880 measured reflections
1935 independent reflections
1474 reflections with *I* > 2σ(*I*)
*R*_int_ = 0.036

#### Refinement

*R*[*F* ^2^ > 2σ(*F* ^2^)] = 0.040
*wR*(*F* ^2^) = 0.116
*S* = 1.09
1935 reflections
147 parameters
H-atom parameters constrained
Δρ_max_ = 0.18 e Å^−3^
Δρ_min_ = −0.19 e Å^−3^

### 

Data collection: *APEX2* (Bruker, 2004 ▶); cell refinement: *SAINT* (Bruker, 2004 ▶); data reduction: *SAINT*; program(s) used to solve structure: *SHELXS97* (Sheldrick, 2008 ▶); program(s) used to refine structure: *SHELXL97* (Sheldrick, 2008 ▶); molecular graphics: *SHELXTL* (Sheldrick, 2008 ▶); software used to prepare material for publication: *SHELXTL*.

## Supplementary Material

Crystal structure: contains datablock(s) global, I. DOI: 10.1107/S1600536811019921/hb5896sup1.cif

Structure factors: contains datablock(s) I. DOI: 10.1107/S1600536811019921/hb5896Isup2.hkl

Supplementary material file. DOI: 10.1107/S1600536811019921/hb5896Isup3.cml

Additional supplementary materials:  crystallographic information; 3D view; checkCIF report

## Figures and Tables

**Table 1:** Hydrogen-bond geometry (Å, °)

*D*—H⋯*A*	*D*—H	H⋯*A*	*D*⋯*A*	*D*—H⋯*A*
O1—H1*A*⋯N1^i^	0.82	2.09	2.885 (2)	163

Symmetry code: (i) 
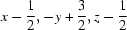
.

**Fig. 1.**
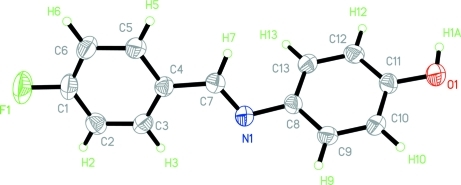
The molecular structure of the title compounds with 50% probability displacement ellipsoids for non-hydrogen atoms.

**Fig. 2.**
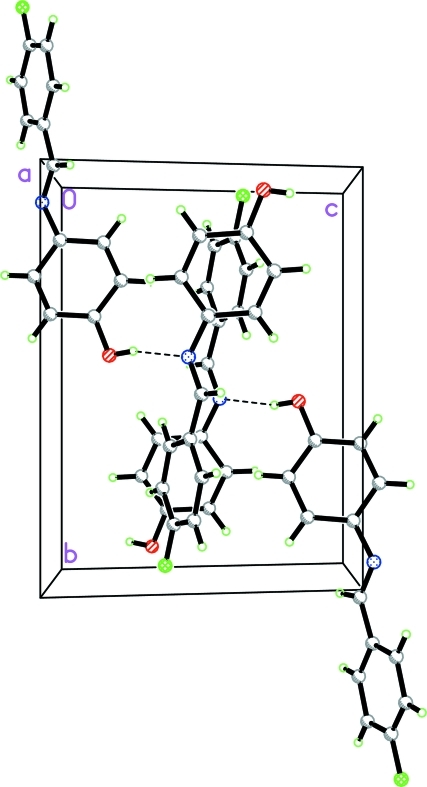
Molecular packing of the title compound, viewed along the a axis. Hydrogen bonds are shown as dashed lines.

## supplementary crystallographic information

### Comment

As part of our ongoing studies of Schiff bases (Sun *et al.*, 2011), we  report here the crystal structure of the title compound, (I). In (I) (Fig. 1), the bond lengths and angles are normal and comparable to the values observed in similar compounds (Nie *et al.*, 2008; Fun *et al.*, 2008; Alhadi *et al.*, 2008). The dihedral angle between the two aromatic rings in the Schiff base molecule is 55.6 °, indicating that two these rings are not coplanar. Intermolecular O-H—N hydrogen bonds (Table 1) link the molecules along *a* axis (Fig. 2).

### Experimental

A mixture of 4-chlorobenzaldehyde (5 mmol), 4-aminophenol (5 mmol) and methanol (40 ml) was refluxed for 2 h. It was then allowed to cool and filtered. Recrystallization of the crude product from methanol yielded yellow blocks of (I).

### Refinement

H atoms were positioned geometrically and refined using the riding-model approximation, with C—H = 0.93–0.97 Å, O—H = 0.82 Å, and *U*_iso_(H) = 1.2*U*_eq_(C) or *U*_iso_(H) = 1.5*U*_eq_(O). In the absence of significant anomalous dispersion effects, Freidel pairs were merged.

### Crystal data

**Table:**

C_13_H_10_FNO	*F*(000) = 448
*M**_r_* = 215.22	*D*_x_ = 1.362 Mg m^−^^3^
Monoclinic, *P*2_1_/*n*	Mo *K*α radiation, λ = 0.71073 Å
Hall symbol: -P 2yn	Cell parameters from 2754 reflections
*a* = 9.400 (5) Å	θ = 2.7–28.2°
*b* = 12.213 (7) Å	µ = 0.10 mm^−^^1^
*c* = 9.450 (5) Å	*T* = 296 K
β = 104.666 (5)°	Block, yellow
*V* = 1049.5 (10) Å^3^	0.25 × 0.23 × 0.22 mm
*Z* = 4	

### Data collection

**Table:**

Bruker APEXII CCD diffractometer	1935 independent reflections
Radiation source: fine-focus sealed tube	1474 reflections with *I* > 2σ(*I*)
graphite	*R*_int_ = 0.036
φ and ω scans	θ_max_ = 25.5°, θ_min_ = 2.7°
Absorption correction: multi-scan (*SADABS*; Bruker, 2004)	*h* = −10→11
*T*_min_ = 0.976, *T*_max_ = 0.979	*k* = −11→14
5880 measured reflections	*l* = −11→11

### Refinement

**Table:**

Refinement on *F*^2^	Secondary atom site location: difference Fourier map
Least-squares matrix: full	Hydrogen site location: inferred from neighbouring sites
*R*[*F*^2^ > 2σ(*F*^2^)] = 0.040	H-atom parameters constrained
*wR*(*F*^2^) = 0.116	*w* = 1/[σ^2^(*F*_o_^2^) + (0.0422*P*)^2^ + 0.3813*P*] where *P* = (*F*_o_^2^ + 2*F*_c_^2^)/3
*S* = 1.09	(Δ/σ)_max_ < 0.001
1935 reflections	Δρ_max_ = 0.18 e Å^−^^3^
147 parameters	Δρ_min_ = −0.19 e Å^−^^3^
0 restraints	Extinction correction: *SHELXL97* (Sheldrick, 2008), Fc^*^=kFc[1+0.001xFc^2^λ^3^/sin(2θ)]^-1/4^
Primary atom site location: structure-invariant direct methods	Extinction coefficient: 0.077 (5)

### Special details

**Table:**

Geometry. All e.s.d.'s (except the e.s.d. in the dihedral angle between two l.s. planes) are estimated using the full covariance matrix. The cell e.s.d.'s are taken into account individually in the estimation of e.s.d.'s in distances, angles and torsion angles; correlations between e.s.d.'s in cell parameters are only used when they are defined by crystal symmetry. An approximate (isotropic) treatment of cell e.s.d.'s is used for estimating e.s.d.'s involving l.s. planes.
Refinement. Refinement of *F*^2^ against ALL reflections. The weighted *R*-factor *wR* and goodness of fit *S* are based on *F*^2^, conventional *R*-factors *R* are based on *F*, with *F* set to zero for negative *F*^2^. The threshold expression of *F*^2^ > σ(*F*^2^) is used only for calculating *R*-factors(gt) *etc*. and is not relevant to the choice of reflections for refinement. *R*-factors based on *F*^2^ are statistically about twice as large as those based on *F*, and *R*- factors based on ALL data will be even larger.

### Fractional atomic coordinates and isotropic or equivalent isotropic displacement parameters (Å^2^)

**Table:**

	*x*	*y*	*z*	*U*_iso_*/*U*_eq_	
C1	0.3906 (2)	0.14260 (14)	0.6005 (2)	0.0440 (5)	
C2	0.4887 (2)	0.22747 (14)	0.63956 (18)	0.0409 (4)	
H2	0.5876	0.2139	0.6826	0.049*	
C3	0.43732 (19)	0.33301 (14)	0.61347 (18)	0.0368 (4)	
H3	0.5019	0.3915	0.6400	0.044*	
C4	0.28916 (18)	0.35297 (12)	0.54763 (17)	0.0327 (4)	
C5	0.1950 (2)	0.26412 (14)	0.50840 (18)	0.0383 (4)	
H5	0.0962	0.2765	0.4636	0.046*	
C6	0.2447 (2)	0.15804 (14)	0.5344 (2)	0.0451 (5)	
H6	0.1812	0.0989	0.5079	0.054*	
C7	0.22867 (19)	0.46193 (13)	0.50988 (18)	0.0355 (4)	
H7	0.1338	0.4662	0.4497	0.043*	
C8	0.22355 (17)	0.65015 (12)	0.48809 (17)	0.0317 (4)	
C9	0.22777 (18)	0.74242 (13)	0.57483 (17)	0.0343 (4)	
H9	0.2750	0.7392	0.6739	0.041*	
C10	0.16262 (19)	0.83889 (13)	0.51566 (18)	0.0373 (4)	
H10	0.1647	0.8998	0.5752	0.045*	
C11	0.09399 (18)	0.84530 (13)	0.36764 (18)	0.0354 (4)	
C12	0.09253 (18)	0.75496 (14)	0.27921 (18)	0.0363 (4)	
H12	0.0489	0.7595	0.1794	0.044*	
C13	0.15602 (19)	0.65777 (13)	0.33919 (18)	0.0362 (4)	
H13	0.1536	0.5969	0.2794	0.043*	
N1	0.29403 (15)	0.55196 (11)	0.55190 (14)	0.0344 (4)	
F1	0.44178 (15)	0.03938 (9)	0.62945 (16)	0.0739 (4)	
O1	0.03080 (16)	0.94277 (10)	0.31471 (14)	0.0520 (4)	
H1A	−0.0279	0.9327	0.2353	0.078*	

### Atomic displacement parameters (Å^2^)

**Table:**

	*U*^11^	*U*^22^	*U*^33^	*U*^12^	*U*^13^	*U*^23^
C1	0.0595 (12)	0.0274 (9)	0.0470 (10)	0.0055 (8)	0.0167 (9)	0.0046 (7)
C2	0.0407 (10)	0.0414 (10)	0.0392 (9)	0.0064 (8)	0.0077 (8)	0.0005 (7)
C3	0.0408 (10)	0.0324 (9)	0.0371 (9)	−0.0032 (7)	0.0096 (7)	−0.0048 (7)
C4	0.0393 (9)	0.0291 (9)	0.0298 (8)	−0.0013 (7)	0.0089 (7)	0.0004 (6)
C5	0.0402 (10)	0.0359 (10)	0.0371 (9)	−0.0045 (7)	0.0064 (7)	0.0029 (7)
C6	0.0550 (12)	0.0302 (10)	0.0494 (10)	−0.0093 (8)	0.0117 (9)	−0.0002 (8)
C7	0.0350 (9)	0.0327 (9)	0.0371 (9)	−0.0010 (7)	0.0061 (7)	0.0017 (7)
C8	0.0314 (9)	0.0279 (9)	0.0346 (8)	−0.0006 (6)	0.0065 (7)	0.0036 (6)
C9	0.0371 (9)	0.0333 (9)	0.0295 (8)	−0.0027 (7)	0.0031 (7)	0.0009 (7)
C10	0.0427 (10)	0.0278 (9)	0.0390 (9)	−0.0022 (7)	0.0060 (7)	−0.0026 (7)
C11	0.0352 (9)	0.0279 (9)	0.0405 (9)	−0.0013 (7)	0.0049 (7)	0.0058 (7)
C12	0.0394 (10)	0.0374 (10)	0.0292 (8)	−0.0013 (7)	0.0033 (7)	0.0025 (7)
C13	0.0410 (10)	0.0318 (9)	0.0344 (9)	−0.0011 (7)	0.0070 (7)	−0.0035 (7)
N1	0.0380 (8)	0.0291 (8)	0.0345 (7)	0.0009 (6)	0.0064 (6)	0.0020 (6)
F1	0.0840 (10)	0.0317 (7)	0.1022 (10)	0.0132 (6)	0.0167 (8)	0.0105 (6)
O1	0.0641 (9)	0.0294 (7)	0.0508 (8)	0.0039 (6)	−0.0071 (6)	0.0060 (5)

### Geometric parameters (Å, °)

**Table:**

C1—F1	1.352 (2)	C8—C9	1.388 (2)
C1—C6	1.368 (3)	C8—C13	1.392 (2)
C1—C2	1.374 (3)	C8—N1	1.428 (2)
C2—C3	1.377 (2)	C9—C10	1.379 (2)
C2—H2	0.9300	C9—H9	0.9300
C3—C4	1.395 (2)	C10—C11	1.386 (2)
C3—H3	0.9300	C10—H10	0.9300
C4—C5	1.390 (2)	C11—O1	1.367 (2)
C4—C7	1.456 (2)	C11—C12	1.382 (2)
C5—C6	1.378 (2)	C12—C13	1.384 (2)
C5—H5	0.9300	C12—H12	0.9300
C6—H6	0.9300	C13—H13	0.9300
C7—N1	1.273 (2)	O1—H1A	0.8200
C7—H7	0.9300		
			
F1—C1—C6	118.99 (17)	C9—C8—C13	118.68 (14)
F1—C1—C2	117.95 (17)	C9—C8—N1	119.45 (14)
C6—C1—C2	123.06 (16)	C13—C8—N1	121.80 (14)
C1—C2—C3	118.47 (17)	C10—C9—C8	120.71 (15)
C1—C2—H2	120.8	C10—C9—H9	119.6
C3—C2—H2	120.8	C8—C9—H9	119.6
C2—C3—C4	120.59 (16)	C9—C10—C11	120.18 (15)
C2—C3—H3	119.7	C9—C10—H10	119.9
C4—C3—H3	119.7	C11—C10—H10	119.9
C5—C4—C3	118.60 (15)	O1—C11—C12	122.48 (15)
C5—C4—C7	117.74 (15)	O1—C11—C10	117.79 (15)
C3—C4—C7	123.56 (15)	C12—C11—C10	119.73 (15)
C6—C5—C4	121.46 (17)	C13—C12—C11	119.99 (15)
C6—C5—H5	119.3	C13—C12—H12	120.0
C4—C5—H5	119.3	C11—C12—H12	120.0
C1—C6—C5	117.80 (16)	C12—C13—C8	120.68 (15)
C1—C6—H6	121.1	C12—C13—H13	119.7
C5—C6—H6	121.1	C8—C13—H13	119.7
N1—C7—C4	125.84 (15)	C7—N1—C8	117.26 (14)
N1—C7—H7	117.1	C11—O1—H1A	109.5
C4—C7—H7	117.1		
			
F1—C1—C2—C3	−178.66 (16)	N1—C8—C9—C10	178.98 (15)
C6—C1—C2—C3	1.3 (3)	C8—C9—C10—C11	−1.1 (3)
C1—C2—C3—C4	−0.6 (3)	C9—C10—C11—O1	179.82 (16)
C2—C3—C4—C5	−0.3 (2)	C9—C10—C11—C12	−0.7 (3)
C2—C3—C4—C7	−176.69 (15)	O1—C11—C12—C13	−178.88 (16)
C3—C4—C5—C6	0.6 (3)	C10—C11—C12—C13	1.7 (3)
C7—C4—C5—C6	177.20 (16)	C11—C12—C13—C8	−0.9 (3)
F1—C1—C6—C5	178.95 (16)	C9—C8—C13—C12	−0.9 (3)
C2—C1—C6—C5	−1.0 (3)	N1—C8—C13—C12	−177.93 (16)
C4—C5—C6—C1	0.0 (3)	C4—C7—N1—C8	172.19 (15)
C5—C4—C7—N1	171.35 (17)	C9—C8—N1—C7	140.76 (16)
C3—C4—C7—N1	−12.2 (3)	C13—C8—N1—C7	−42.2 (2)
C13—C8—C9—C10	1.9 (3)		

### Hydrogen-bond geometry (Å, °)

**Table:**

*D*—H···*A*	*D*—H	H···*A*	*D*···*A*	*D*—H···*A*
O1—H1A···N1^i^	0.82	2.09	2.885 (2)	163

Symmetry codes: (i) *x*−1/2, −*y*+3/2, *z*−1/2.
